# Aqua­(2-hydrazino-1,10-phenanthroline)nitratocopper(II) nitrate

**DOI:** 10.1107/S1600536808015523

**Published:** 2008-05-30

**Authors:** Hong Liang Li, Qi Sheng Liu

**Affiliations:** a Department of Chemistry, Dezhou University, Dezhou Shandong 253023, People’s Republic of China; b Department of Chemistry, Shandong Normal University, Jinan 250014, People’s Republic of China

## Abstract

In the title mononuclear complex, [Cu(NO_3_)(C_12_H_10_N_4_)(H_2_O)]NO_3_, the Cu^II^ ion assumes a distorted square-pyramidal geometry. There is a π–π stacking inter­action between the five-membered ring containing the Cu atom and a pyridine ring of a neighboring complex [centroid–centroid distance = 3.567 (2) Å and a perpendicular distance of 3.394 Å]. The crystal structure also contains inter­molecular N—H⋯O, O—H⋯O and C—H⋯O hydrogen bonds, linking cations and anions. In addition, there is a short inter­molecular contact [2.784 (6) Å] between an O atom of the coordinated nitrate group and its symmetry-related atom.

## Related literature

For related structures, see: Liu *et al.* (2008 ▶); Lewis *et al.* (1980 ▶).
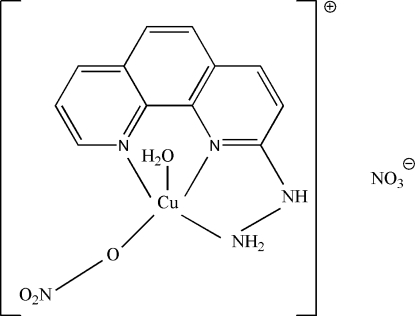


## Experimental

### 

#### Crystal data

[Cu(NO_3_)(C_12_H_10_N_4_)(H_2_O)]NO_3_
*M* *_r_* = 415.82
Monoclinic, 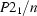

*a* = 8.7175 (8) Å
*b* = 10.7746 (10) Å
*c* = 16.4725 (16) Å
β = 97.175 (2)°
*V* = 1535.1 (2) Å^3^
*Z* = 4
Mo *K*α radiation
μ = 1.48 mm^−1^
*T* = 298 (2) K
0.50 × 0.20 × 0.12 mm

#### Data collection

Bruker SMART APEX CCD diffractometer
Absorption correction: multi-scan (*SADABS*; Sheldrick, 1996 ▶) *T*_min_ = 0.525, *T*_max_ = 0.843
8857 measured reflections
3329 independent reflections
2735 reflections with *I* > 2σ(*I*)
*R*_int_ = 0.034

#### Refinement

*R*[*F* ^2^ > 2σ(*F* ^2^)] = 0.045
*wR*(*F* ^2^) = 0.112
*S* = 1.03
3329 reflections
235 parameters
3 restraints
H-atom parameters constrained
Δρ_max_ = 0.70 e Å^−3^
Δρ_min_ = −0.33 e Å^−3^

### 

Data collection: *SMART* (Bruker, 1997 ▶); cell refinement: *SAINT* (Bruker, 1997 ▶); data reduction: *SAINT*; program(s) used to solve structure: *SHELXTL* (Sheldrick, 2008 ▶); program(s) used to refine structure: *SHELXTL*; molecular graphics: *SHELXTL*; software used to prepare material for publication: *SHELXTL* and local programs.

## Supplementary Material

Crystal structure: contains datablocks I, global. DOI: 10.1107/S1600536808015523/wn2264sup1.cif

Structure factors: contains datablocks I. DOI: 10.1107/S1600536808015523/wn2264Isup2.hkl

Additional supplementary materials:  crystallographic information; 3D view; checkCIF report

## Figures and Tables

**Table 1:** Hydrogen-bond geometry (Å, °)

*D*—H⋯*A*	*D*—H	H⋯*A*	*D*⋯*A*	*D*—H⋯*A*
N1—H1⋯O6^i^	0.86	2.18	2.936 (4)	146
N1—H1⋯O7^i^	0.86	2.54	3.181 (4)	132
N2—H2*A*⋯O3^ii^	0.90	2.22	3.111 (4)	169
N2—H2*B*⋯O7^iii^	0.90	2.17	3.055 (4)	168
O1—H9⋯O5^iii^	0.84	1.98	2.818 (3)	175
O1—H9⋯O7^iii^	0.84	2.58	3.185 (3)	130
O1—H13⋯O6^iv^	0.85	1.99	2.821 (3)	167
C2—H2⋯O6^i^	0.93	2.56	3.253 (4)	132
C3—H3⋯O1^v^	0.93	2.48	3.280 (4)	144
C11—H11⋯O4^vi^	0.93	2.43	3.118 (5)	131

Symmetry codes: (i) 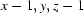; (ii) 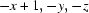; (iii) 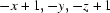; (iv) 
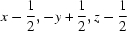
; (v) 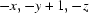; (vi) 
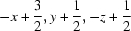
.

**Fig. 1.**
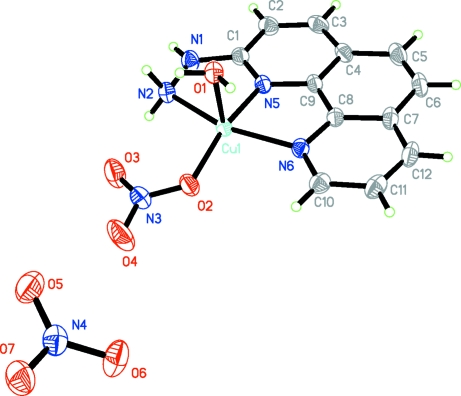
Structure of the title complex with the atom-numbering scheme. Displacement ellipsoids are drawn at the 30% probability level.

**Fig. 2.**
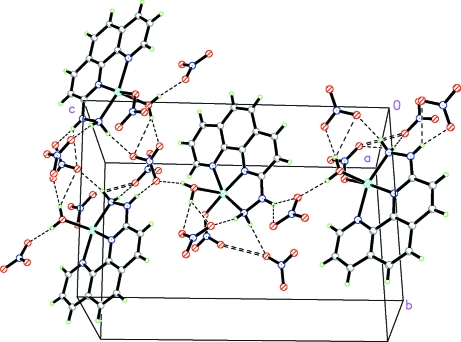
A view of the packing in the crystal structure. Short contacts between atom O3 and its symmetry-related atoms are shown as double dashed lines and hydrogen bonds as dashed lines.

## supplementary crystallographic information

### Comment

Derivatives of 1,10-phenanthroline play an important role in modern coordination chemistry (Liu *et al.*, 2008), and although complexes with 2,9-dihydrazino-1,10-phenanthroline as ligand have been published (Lewis *et al.*, 1980), to the best of our knowledge, no crystal structure of the title complex has been published.

Fig. 1 shows the structure, revealing that the Cu atom is in a distorted square-pyramidal environment, with atom O1 in the apical position. There is a single π-π stacking interaction involving symmetry-related 1,10-phenanthroline ligands, the relevant distances being *Cg*1···*Cg*2^v^ = 3.567 (2) Å and *Cg*1···*Cg*2^v^_perp_ = 3.394 Å and α = 3.76° [symmetry code: (v) -*x*, 1 - *y*, -*z*; *Cg*1 and *Cg*2 are the centroids of the Cu1/N5/N6/C8/C9 ring and N6/C7/C8/C10-C12 ring, respectively; *Cg*1···*Cg*2^1^_perp_ is the perpendicular distance from ring *Cg*1 to ring *Cg*2^i^; α is the dihedral angle between ring plane *Cg*1 and ring plane *Cg*2^i^]. There exists a short contact [2.784 (6) Å] between atom O3 and its symmetry-related atom O3^ii^ [symmetry code: (ii) 1-*x*,-*y*,-*z*], as shown in Fig. 2 (double dashed lines). In addition, the crystal structure contains classical N—H..O and O—H···O hydrogen bonds, also non-classical C—H···O hydrogen bonds, as shown in Table 1 and Fig. 2. The π-π stacking interaction, the short contact between atom O3 and its symmetry-related atom O3^ii^ and the hydrogen bonds stabilize the crystal structure.

### Experimental

10 ml methanol solution of 2-hydrazino-1,10-phenanthroline (0.0105 g, 0.0576 mmol) was added to 5 ml aqueous solution of Cu(NO_3_)_2_.3H_2_O (0.0390 g, 0.161 mmol) and the mixture was stirred for a few minutes. Deep-green single crystals were obtained after the filtrate had been allowed to stand at room temperature for two weeks.

### Refinement

Oxygen-bound H atoms were located in a difference Fourier map, then placed in calculated positions with O—H = 0.84 and 0.85 Å and refined as riding with *U*_iso_(H) = 1.5*U*_eq_(O). Other H atoms were placed in calculated positions with C—H = 0.93 Å and N—H = 0.86 and 0.90 Å, and refined as riding with *U*_iso_(H) = 1.2*U*_eq_(C,N).

### Crystal data

**Table:**

[Cu(NO_3_)(C_12_H_10_N_4_)(H_2_O)]NO_3_	*F*_000_ = 844
*M**_r_* = 415.82	*D*_x_ = 1.799 Mg m^−^^3^
Monoclinic, *P*2_1_/*n*	Mo *K*α radiation λ = 0.71073 Å
Hall symbol: -P 2yn	Cell parameters from 2442 reflections
*a* = 8.7175 (8) Å	θ = 2.3–24.6º
*b* = 10.7746 (10) Å	µ = 1.48 mm^−^^1^
*c* = 16.4725 (16) Å	*T* = 298 (2) K
β = 97.175 (2)º	Block, green
*V* = 1535.1 (2) Å^3^	0.50 × 0.20 × 0.12 mm
*Z* = 4	

### Data collection

**Table:**

Bruker SMART APEX CCD diffractometer	3329 independent reflections
Radiation source: fine-focus sealed tube	2735 reflections with *I* > 2σ(*I*)
Monochromator: graphite	*R*_int_ = 0.034
*T* = 298(2) K	θ_max_ = 27.0º
φ and ω scans	θ_min_ = 2.3º
Absorption correction: multi-scan(SADABS; Sheldrick, 1996)	*h* = −11→11
*T*_min_ = 0.525, *T*_max_ = 0.843	*k* = −13→13
8857 measured reflections	*l* = −12→20

### Refinement

**Table:**

Refinement on *F*^2^	Secondary atom site location: difference Fourier map
Least-squares matrix: full	Hydrogen site location: inferred from neighbouring sites
*R*[*F*^2^ > 2σ(*F*^2^)] = 0.045	H-atom parameters constrained
*wR*(*F*^2^) = 0.112	*w* = 1/[σ^2^(*F*_o_^2^) + (0.058*P*)^2^ + 0.4576*P*] where *P* = (*F*_o_^2^ + 2*F*_c_^2^)/3
*S* = 1.03	(Δ/σ)_max_ = 0.002
3329 reflections	Δρ_max_ = 0.70 e Å^−^^3^
235 parameters	Δρ_min_ = −0.33 e Å^−^^3^
3 restraints	Extinction correction: none
Primary atom site location: structure-invariant direct methods	

### Special details

**Table:**

Geometry. All e.s.d.'s (except the e.s.d. in the dihedral angle between two l.s. planes) are estimated using the full covariance matrix. The cell e.s.d.'s are taken into account individually in the estimation of e.s.d.'s in distances, angles and torsion angles; correlations between e.s.d.'s in cell parameters are only used when they are defined by crystal symmetry. An approximate (isotropic) treatment of cell e.s.d.'s is used for estimating e.s.d.'s involving l.s. planes.
Refinement. Refinement of *F*^2^ against ALL reflections. The weighted *R*-factor *wR* and goodness of fit *S* are based on *F*^2^, conventional *R*-factors *R* are based on *F*, with *F* set to zero for negative *F*^2^. The threshold expression of *F*^2^ > σ(*F*^2^) is used only for calculating *R*-factors(gt) *etc*. and is not relevant to the choice of reflections for refinement. *R*-factors based on *F*^2^ are statistically about twice as large as those based on *F*, and *R*- factors based on ALL data will be even larger.

### Fractional atomic coordinates and isotropic or equivalent isotropic displacement parameters (Å^2^)

**Table:**

	*x*	*y*	*z*	*U*_iso_*/*U*_eq_	
C1	0.1553 (4)	0.3275 (3)	−0.07122 (17)	0.0395 (7)	
C2	0.0545 (4)	0.3975 (3)	−0.12818 (19)	0.0478 (8)	
H2	−0.0080	0.3581	−0.1704	0.057*	
C3	0.0507 (4)	0.5226 (3)	−0.1201 (2)	0.0492 (8)	
H3	−0.0159	0.5689	−0.1568	0.059*	
C4	0.1460 (4)	0.5839 (3)	−0.05691 (19)	0.0441 (8)	
C5	0.1525 (5)	0.7152 (3)	−0.0411 (2)	0.0539 (9)	
H5	0.0886	0.7683	−0.0745	0.065*	
C6	0.2498 (4)	0.7624 (3)	0.0215 (2)	0.0541 (9)	
H6	0.2504	0.8477	0.0302	0.065*	
C7	0.3518 (4)	0.6866 (3)	0.0746 (2)	0.0459 (8)	
C8	0.3481 (3)	0.5572 (3)	0.06060 (17)	0.0377 (7)	
C9	0.2440 (3)	0.5098 (3)	−0.00470 (18)	0.0364 (6)	
C10	0.5401 (4)	0.5172 (3)	0.1667 (2)	0.0462 (8)	
H10	0.6058	0.4620	0.1974	0.055*	
C11	0.5497 (4)	0.6440 (3)	0.1859 (2)	0.0550 (9)	
H11	0.6190	0.6713	0.2297	0.066*	
C12	0.4578 (5)	0.7272 (3)	0.1405 (2)	0.0548 (9)	
H12	0.4653	0.8113	0.1533	0.066*	
Cu1	0.37741 (4)	0.29261 (3)	0.06801 (2)	0.03598 (14)	
N1	0.1698 (3)	0.2036 (2)	−0.07207 (16)	0.0478 (7)	
H1	0.1211	0.1587	−0.1101	0.057*	
N2	0.2703 (3)	0.1510 (2)	−0.00644 (15)	0.0432 (6)	
H2A	0.3427	0.1045	−0.0265	0.052*	
H2B	0.2160	0.1014	0.0235	0.052*	
N3	0.5836 (3)	0.1007 (3)	0.13379 (17)	0.0499 (7)	
N4	0.8613 (3)	0.0304 (2)	0.83090 (17)	0.0472 (7)	
N5	0.2458 (3)	0.3843 (2)	−0.01228 (14)	0.0362 (5)	
N6	0.4397 (3)	0.4735 (2)	0.10578 (15)	0.0376 (5)	
O1	0.2264 (3)	0.27432 (18)	0.16726 (12)	0.0440 (5)	
H9	0.2273	0.1980	0.1784	0.066*	
H13	0.2717	0.3128	0.2083	0.066*	
O2	0.5464 (3)	0.21509 (19)	0.13917 (15)	0.0516 (6)	
O3	0.5139 (4)	0.0349 (2)	0.08196 (18)	0.0770 (9)	
O4	0.6890 (4)	0.0598 (3)	0.1787 (2)	0.1068 (13)	
O5	0.7508 (3)	−0.0211 (2)	0.79020 (18)	0.0735 (8)	
O6	0.9063 (3)	0.1324 (2)	0.80827 (14)	0.0610 (7)	
O7	0.9290 (3)	−0.0178 (2)	0.89371 (16)	0.0627 (7)	

### Atomic displacement parameters (Å^2^)

**Table:**

	*U*^11^	*U*^22^	*U*^33^	*U*^12^	*U*^13^	*U*^23^
C1	0.0392 (17)	0.0383 (16)	0.0423 (17)	0.0019 (13)	0.0106 (13)	0.0020 (13)
C2	0.0441 (19)	0.052 (2)	0.0457 (18)	0.0053 (15)	0.0008 (14)	0.0062 (15)
C3	0.0418 (19)	0.057 (2)	0.0492 (19)	0.0118 (16)	0.0085 (15)	0.0152 (16)
C4	0.0469 (19)	0.0369 (16)	0.0527 (19)	0.0104 (14)	0.0224 (15)	0.0106 (13)
C5	0.063 (2)	0.0395 (18)	0.062 (2)	0.0131 (16)	0.0210 (18)	0.0150 (16)
C6	0.071 (3)	0.0282 (16)	0.069 (2)	0.0072 (16)	0.034 (2)	0.0047 (15)
C7	0.058 (2)	0.0293 (15)	0.056 (2)	−0.0025 (14)	0.0284 (17)	−0.0018 (13)
C8	0.0413 (17)	0.0320 (15)	0.0436 (16)	−0.0005 (12)	0.0199 (13)	0.0007 (12)
C9	0.0404 (17)	0.0297 (14)	0.0416 (16)	0.0044 (12)	0.0149 (13)	0.0045 (12)
C10	0.0457 (19)	0.0491 (18)	0.0451 (17)	−0.0050 (15)	0.0115 (14)	−0.0034 (14)
C11	0.060 (2)	0.054 (2)	0.053 (2)	−0.0171 (18)	0.0127 (17)	−0.0129 (17)
C12	0.070 (3)	0.0332 (17)	0.066 (2)	−0.0128 (16)	0.0264 (19)	−0.0120 (15)
Cu1	0.0392 (2)	0.0291 (2)	0.0397 (2)	0.00421 (14)	0.00535 (15)	0.00074 (14)
N1	0.0554 (17)	0.0373 (14)	0.0479 (16)	0.0027 (12)	−0.0051 (13)	−0.0059 (11)
N2	0.0515 (17)	0.0317 (13)	0.0464 (14)	0.0037 (11)	0.0065 (12)	−0.0001 (11)
N3	0.0537 (18)	0.0477 (16)	0.0474 (15)	0.0124 (14)	0.0022 (13)	0.0019 (13)
N4	0.0568 (18)	0.0386 (14)	0.0483 (16)	0.0009 (13)	0.0148 (13)	0.0022 (12)
N5	0.0394 (14)	0.0306 (12)	0.0390 (13)	0.0036 (10)	0.0061 (10)	0.0011 (10)
N6	0.0409 (14)	0.0326 (12)	0.0412 (13)	−0.0001 (11)	0.0132 (11)	−0.0014 (11)
O1	0.0505 (14)	0.0375 (11)	0.0447 (12)	0.0008 (9)	0.0091 (10)	0.0001 (9)
O2	0.0519 (15)	0.0406 (12)	0.0600 (14)	0.0120 (10)	−0.0020 (11)	−0.0063 (10)
O3	0.095 (2)	0.0483 (14)	0.0791 (19)	0.0180 (14)	−0.0225 (16)	−0.0118 (14)
O4	0.108 (3)	0.077 (2)	0.119 (3)	0.042 (2)	−0.051 (2)	−0.0025 (19)
O5	0.0673 (18)	0.0551 (15)	0.092 (2)	−0.0193 (14)	−0.0152 (15)	0.0131 (14)
O6	0.0885 (19)	0.0425 (13)	0.0500 (13)	−0.0200 (13)	0.0008 (12)	0.0065 (11)
O7	0.0719 (18)	0.0546 (15)	0.0601 (15)	0.0045 (13)	0.0018 (13)	0.0167 (12)

### Geometric parameters (Å, °)

**Table:**

C1—N5	1.322 (4)	C11—C12	1.362 (5)
C1—N1	1.341 (4)	C11—H11	0.9300
C1—C2	1.420 (4)	C12—H12	0.9300
C2—C3	1.356 (5)	Cu1—N5	1.914 (2)
C2—H2	0.9300	Cu1—O2	1.952 (2)
C3—C4	1.412 (5)	Cu1—N6	2.097 (2)
C3—H3	0.9300	Cu1—N2	2.102 (2)
C4—C9	1.387 (4)	Cu1—O1	2.232 (2)
C4—C5	1.437 (5)	N1—N2	1.422 (3)
C5—C6	1.351 (5)	N1—H1	0.8600
C5—H5	0.9300	N2—H2A	0.9000
C6—C7	1.425 (5)	N2—H2B	0.9000
C6—H6	0.9300	N3—O4	1.190 (4)
C7—C12	1.405 (5)	N3—O3	1.213 (4)
C7—C8	1.412 (4)	N3—O2	1.280 (3)
C8—N6	1.363 (4)	N4—O5	1.234 (3)
C8—C9	1.413 (4)	N4—O7	1.239 (3)
C9—N5	1.358 (4)	N4—O6	1.240 (3)
C10—N6	1.332 (4)	O1—H9	0.8422
C10—C11	1.402 (5)	O1—H13	0.8485
C10—H10	0.9300		
			
N5—C1—N1	114.9 (3)	N5—Cu1—O2	168.02 (11)
N5—C1—C2	120.2 (3)	N5—Cu1—N6	80.55 (10)
N1—C1—C2	125.0 (3)	O2—Cu1—N6	94.10 (9)
C3—C2—C1	118.9 (3)	N5—Cu1—N2	77.72 (10)
C3—C2—H2	120.5	O2—Cu1—N2	106.66 (9)
C1—C2—H2	120.5	N6—Cu1—N2	158.07 (10)
C2—C3—C4	121.3 (3)	N5—Cu1—O1	101.19 (9)
C2—C3—H3	119.4	O2—Cu1—O1	89.57 (10)
C4—C3—H3	119.4	N6—Cu1—O1	91.11 (8)
C9—C4—C3	116.6 (3)	N2—Cu1—O1	95.94 (9)
C9—C4—C5	116.6 (3)	C1—N1—N2	116.0 (2)
C3—C4—C5	126.9 (3)	C1—N1—H1	122.0
C6—C5—C4	121.0 (3)	N2—N1—H1	122.0
C6—C5—H5	119.5	N1—N2—Cu1	109.92 (17)
C4—C5—H5	119.5	N1—N2—H2A	109.7
C5—C6—C7	122.5 (3)	Cu1—N2—H2A	109.7
C5—C6—H6	118.8	N1—N2—H2B	109.7
C7—C6—H6	118.8	Cu1—N2—H2B	109.7
C12—C7—C8	115.7 (3)	H2A—N2—H2B	108.2
C12—C7—C6	126.6 (3)	O4—N3—O3	120.0 (3)
C8—C7—C6	117.8 (3)	O4—N3—O2	119.7 (3)
N6—C8—C7	124.3 (3)	O3—N3—O2	120.2 (3)
N6—C8—C9	117.0 (3)	O5—N4—O7	121.6 (3)
C7—C8—C9	118.7 (3)	O5—N4—O6	119.3 (3)
N5—C9—C4	122.0 (3)	O7—N4—O6	119.1 (3)
N5—C9—C8	114.6 (3)	C1—N5—C9	121.1 (3)
C4—C9—C8	123.4 (3)	C1—N5—Cu1	121.3 (2)
N6—C10—C11	121.9 (3)	C9—N5—Cu1	117.6 (2)
N6—C10—H10	119.0	C10—N6—C8	117.6 (3)
C11—C10—H10	119.0	C10—N6—Cu1	132.3 (2)
C12—C11—C10	120.2 (3)	C8—N6—Cu1	109.92 (19)
C12—C11—H11	119.9	Cu1—O1—H9	104.8
C10—C11—H11	119.9	Cu1—O1—H13	106.4
C11—C12—C7	120.2 (3)	H9—O1—H13	108.2
C11—C12—H12	119.9	N3—O2—Cu1	123.3 (2)
C7—C12—H12	119.9		
			
N5—C1—C2—C3	−1.2 (5)	N1—C1—N5—Cu1	−3.2 (4)
N1—C1—C2—C3	179.7 (3)	C2—C1—N5—Cu1	177.6 (2)
C1—C2—C3—C4	0.7 (5)	C4—C9—N5—C1	1.2 (4)
C2—C3—C4—C9	0.7 (5)	C8—C9—N5—C1	−179.4 (3)
C2—C3—C4—C5	−179.4 (3)	C4—C9—N5—Cu1	−176.2 (2)
C9—C4—C5—C6	0.2 (5)	C8—C9—N5—Cu1	3.2 (3)
C3—C4—C5—C6	−179.7 (3)	O2—Cu1—N5—C1	113.5 (5)
C4—C5—C6—C7	0.5 (5)	N6—Cu1—N5—C1	177.7 (2)
C5—C6—C7—C12	179.3 (3)	N2—Cu1—N5—C1	0.7 (2)
C5—C6—C7—C8	−0.5 (5)	O1—Cu1—N5—C1	−93.0 (2)
C12—C7—C8—N6	−0.5 (4)	O2—Cu1—N5—C9	−69.0 (5)
C6—C7—C8—N6	179.3 (3)	N6—Cu1—N5—C9	−4.8 (2)
C12—C7—C8—C9	180.0 (3)	N2—Cu1—N5—C9	178.2 (2)
C6—C7—C8—C9	−0.2 (4)	O1—Cu1—N5—C9	84.5 (2)
C3—C4—C9—N5	−1.7 (4)	C11—C10—N6—C8	1.7 (5)
C5—C4—C9—N5	178.4 (3)	C11—C10—N6—Cu1	−172.6 (2)
C3—C4—C9—C8	179.0 (3)	C7—C8—N6—C10	−0.6 (4)
C5—C4—C9—C8	−0.9 (4)	C9—C8—N6—C10	178.9 (3)
N6—C8—C9—N5	2.0 (4)	C7—C8—N6—Cu1	174.9 (2)
C7—C8—C9—N5	−178.4 (3)	C9—C8—N6—Cu1	−5.5 (3)
N6—C8—C9—C4	−178.6 (3)	N5—Cu1—N6—C10	−179.9 (3)
C7—C8—C9—C4	0.9 (4)	O2—Cu1—N6—C10	−10.7 (3)
N6—C10—C11—C12	−1.8 (5)	N2—Cu1—N6—C10	−172.0 (3)
C10—C11—C12—C7	0.6 (5)	O1—Cu1—N6—C10	79.0 (3)
C8—C7—C12—C11	0.5 (5)	N5—Cu1—N6—C8	5.51 (18)
C6—C7—C12—C11	−179.3 (3)	O2—Cu1—N6—C8	174.71 (19)
N5—C1—N1—N2	4.7 (4)	N2—Cu1—N6—C8	13.3 (4)
C2—C1—N1—N2	−176.2 (3)	O1—Cu1—N6—C8	−95.65 (19)
C1—N1—N2—Cu1	−4.0 (3)	O4—N3—O2—Cu1	179.2 (3)
N5—Cu1—N2—N1	1.71 (19)	O3—N3—O2—Cu1	0.9 (5)
O2—Cu1—N2—N1	−166.8 (2)	N5—Cu1—O2—N3	−105.8 (5)
N6—Cu1—N2—N1	−6.2 (4)	N6—Cu1—O2—N3	−168.8 (3)
O1—Cu1—N2—N1	101.9 (2)	N2—Cu1—O2—N3	4.1 (3)
N1—C1—N5—C9	179.4 (3)	O1—Cu1—O2—N3	100.2 (3)
C2—C1—N5—C9	0.3 (4)		

### Hydrogen-bond geometry (Å, °)

**Table:**

*D*—H···*A*	*D*—H	H···*A*	*D*···*A*	*D*—H···*A*
N1—H1···O6^i^	0.86	2.18	2.936 (4)	146
N1—H1···O7^i^	0.86	2.54	3.181 (4)	132
N2—H2A···O3^ii^	0.90	2.22	3.111 (4)	169
N2—H2B···O7^iii^	0.90	2.17	3.055 (4)	168
O1—H9···O5^iii^	0.84	1.98	2.818 (3)	175
O1—H9···O7^iii^	0.84	2.58	3.185 (3)	130
O1—H13···O6^iv^	0.85	1.99	2.821 (3)	167
C2—H2···O6^i^	0.93	2.56	3.253 (4)	132
C3—H3···O1^v^	0.93	2.48	3.280 (4)	144
C11—H11···O4^vi^	0.93	2.43	3.118 (5)	131

Symmetry codes: (i) *x*−1, *y*, *z*−1; (ii) −*x*+1, −*y*, −*z*; (iii) −*x*+1, −*y*, −*z*+1; (iv) *x*−1/2, −*y*+1/2, *z*−1/2; (v) −*x*, −*y*+1, −*z*; (vi) −*x*+3/2, *y*+1/2, −*z*+1/2.
